# Polyamines Are Present in Mast Cell Secretory Granules and Are Important for Granule Homeostasis

**DOI:** 10.1371/journal.pone.0015071

**Published:** 2010-11-30

**Authors:** Gianni García-Faroldi, Carlos E. Rodríguez, José L. Urdiales, José M. Pérez-Pomares, José C. Dávila, Gunnar Pejler, Francisca Sánchez-Jiménez, Ignacio Fajardo

**Affiliations:** 1 Department of Molecular Biology and Biochemistry, and CIBER de Enfermedades Raras (CIBER-ER), Faculty of Sciences, University of Málaga, Málaga, Spain; 2 Department of Animal Biology, Faculty of Sciences, University of Málaga, Málaga, Spain; 3 Department of Cell Biology, Genetics and Physiology, Faculty of Sciences, University of Málaga, Málaga, Spain; 4 Department of Anatomy, Physiology and Biochemistry, Swedish University of Agricultural Sciences, Uppsala Biomedical Centre (BMC), Uppsala, Sweden; Cornell University, United States of America

## Abstract

**Background:**

Mast cell secretory granules accommodate a large number of components, many of which interact with highly sulfated serglycin proteoglycan (PG) present within the granules. Polyamines (putrescine, spermidine and spermine) are absolutely required for the survival of the vast majority of living cells. Given the reported ability of polyamines to interact with PGs, we investigated the possibility that polyamines may be components of mast cell secretory granules.

**Methodology/Principal Findings:**

Spermidine was released by mouse bone marrow derived mast cells (BMMCs) after degranulation induced by IgE/anti-IgE or calcium ionophore A23187. Additionally, both spermidine and spermine were detected in isolated mouse mast cell granules. Further, depletion of polyamines by culturing BMMCs with α-difluoromethylornithine (DFMO) caused aberrant secretory granule ultrastructure, impaired histamine storage, reduced serotonin levels and increased β-hexosaminidase content. A proteomic approach revealed that DFMO-induced polyamine depletion caused an alteration in the levels of a number of proteins, many of which are connected either with the regulated exocytosis or with the endocytic system.

**Conclusions/Significance:**

Taken together, our results show evidence that polyamines are present in mast cell secretory granules and, furthermore, indicate an essential role of these polycations during the biogenesis and homeostasis of these organelles.

## Introduction

Mast cells participate both in the innate and adaptive immune response [1] and are thereby considered an important component of the immune system. However, mast cells are best known for their implication in a large number of physio-pathological settings, including allergy, asthma, atherosclerosis, inflammatory arthritis, multiple sclerosis and cancer [2]–[7]. The ability of mast cells to take part in all of these processes is mainly attributable to the release of a vast array of molecules (referred to as inflammatory mediators). Among the inflammatory mediators released by mast cells, many are preformed and stored in secretory granules, and can be discharged upon exposure to a number of different stimuli such as crosslinking of IgE bound to the high affinity IgE receptor (FcεRI) [8]. These mediators include biogenic amines such as histamine and serotonin, several mast cell-specific neutral proteases (mainly tryptases and chymases) and cytokines [9]. In addition, essential components of mast cell granules are proteoglycans (PGs), consisting of protein cores to which unbranched, sulfated, and thereby negatively charged polysaccharides of glycosaminoglycan type are attached [8], [10]. The PGs located in mast cell granules are mainly of the serglycin type, and these exert a key role in promoting the storage of numerous mediators, including histamine, serotonin and several mast cell proteases [11]–[16].

Histamine and serotonin are 1,4/1,3-diamines synthesized by amino acid decarboxylases. Other 1,4/1,3-amines are the ornithine-derived polyamines (putrescine, spermidine and spermine), small polycations present in the majority of living organisms where they are absolutely necessary for cell survival. To maintain accurate intracellular concentrations of polyamines, a number of metabolic enzymes and transporters act in concert. Among the enzymes involved in polyamine biosynthesis, it is widely recognized that ornithine decarboxylase (ODC; EC 4.1.1.17) is the key component, its expression and activity being subject to an extraordinary extent of regulation [17], [18]. Polyamines have been implicated in a large number of cellular processes, including functioning of ion channels, nucleic acid packaging, DNA replication, apoptosis, transcription and translation [19]. It is thought that the contribution of polyamines to these processes is mainly explained by their ability to stabilize negatively charged biomolecules such as nucleic acids, proteins and cell membranes [17], [18], [20]. Additional negatively charged macromolecules with the potential to interact with polyamines are the PGs. In fact, a strong interaction of spermine with PGs containing glycosaminoglycans of heparan sulphate/heparin type has been documented [21]. Furthermore, the uptake of polyamines from the extracellular medium depends on their interaction with surface heparan sulfate PGs [22], and it has been demonstrated that glypican-1 is an important PG species mediating this uptake in mammalian cells [23].

Taking into account the established role of polyamines in the packaging of negatively charged macromolecules such as DNA in the nucleus, together with the known interaction of polyamines with PGs, we hypothesized that polyamines might be present in the PG-rich mast cell granules. Indeed, we here present evidence that polyamines are associated with mast cell granules. Furthermore, we demonstrate that polyamines are important for the correct biogenesis and homeostasis of mast cell secretory granules, including proper storage of histamine.

## Materials and Methods

### Ethics statement

This research study was conducted according to regional and national regulations (“Decreto 142/2002” and “Real Decreto 1201/2005”, respectively) and international guidelines (the European Convention and the European Commissions Directive 86/609/EEC on protection of animals used for experimental and other scientific purposes). The study was specifically approved by the Experimentation Ethics Committee of the University of Málaga (CEUMA), Málaga, Spain (procedure code 2009–0054). Mice were kept according to standard routines at the University Animal Center, and were sacrificed by cervical dislocation (a method included in the procedure mentioned above) before obtention of bone marrow derived mast cells (BMMCs).

### Materials

Cell culture media, fetal bovine serum, antibiotics and L-glutamine were purchased from BioWhittaker (Cambrex, UK). Plastics for cell culture were supplied by NUNC (Roskilde, Denmark). Polyclonal antibodies against mouse mast cell protease (mMCP)-6 and mouse MC carboxypeptidase A (mMC-CPA) were raised in rabbits [24], [25]. Rabbit polyclonal antibodies against mannose-6-phosphate receptor binding protein 1 (M6PRBP1) and cytochrome C oxidase (COX)-IV were from Abcam (Cambridge, UK). Rabbit polyclonal antibody against acetyl-histone H3 was from Upstate (Temecula, CA, USA). Mouse monoclonal antibody against β-actin (clone AC-15) was from Sigma-Aldrich (St. Louis, Mo., USA). Monoclonal purified anti-TNP mouse IgE (clone C38-2) and monoclonal purified rat anti-mouse IgE (clone R35-72) were from BD Biosciences Pharmingen (USA). L-[U-^14^C]histidine (50 µCi/mL, >300 mCi/mmol) and Percoll were supplied by GE Healthcare (Chalfont St. Giles, UK). α-Difluoromethylornithine (DFMO) was a kind gift of Dr. Patrick Woster (Wayne State University) who obtained it from Ilex Oncology (San Antonio, Texas). Acetonitrile and methanol for HPLC were from Romil (Teknokroma, Spain). All buffer salts and other chemicals were purchased either from Merck (VWR International) or Sigma-Aldrich.

### Cell culture

BMMCs were obtained essentially as previously described [24]. Briefly, C57BL/6 mice (female, 9–11 weeks old) were sacrificed by cervical dislocation (a method approved by the local ethics committee as stated above), and femur and tibia bone marrow cells were extracted and cultured at 37°C in a humidified atmosphere containing 5% CO_2_ in 50% DMEM supplemented with 10% heat-inactivated fetal bovine serum, 50 µg/mL gentamycin sulphate and 2 mM L-glutamine and 50% WEHI-3B conditioned medium (as a source of IL-3). The cells were kept at a density of 0.5×10^6^ cells/mL, and the culture medium was changed every third day. Mast cell differentiation and maturation was judged by either toluidine blue or May-Grünwald/Giemsa staining, FcεRI and CD117 (c-kit) surface expression, histamine content, mMCP-6 expression and the ability of the cells to degranulate upon stimulation with IgE/anti-IgE. Unless otherwise indicated, all the experiments carried out in the present study were conducted with 3 weeks old BMMCs, where the cell population consisted of more than 90% mast cells. In some experiments, BMMCs were obtained in the presence of DFMO, a treatment used to cause a depletion in polyamines. In this case, bone marrow cells were extracted and cultured as mentioned above and, on day 4, 5 mM DFMO was added to the cell culture medium and maintained for the rest of the culture time. As a control for the effects observed upon addition of DFMO, in some experiments cells were also grown in the presence of DFMO and 100 µM putrescine. This concentration of putrescine was determined as being the optimal in restoring spermidine levels to similar values to those of untreated control cells.

WEHI-3B-conditioned medium was produced by seeding WEHI-3B cells (0.5×10^6^ cells/mL) into the medium described above and incubating them for 3 days.

### Cell staining

Cells were cytospun onto slides and stained using a modified May-Grünwald/Giemsa protocol. Briefly, after fixation in methanol for 3 min, slides were stained for 5 min in May-Grünwald (Fluka), 1 min in 1∶1 dilution of May-Grünwald and 15 min in Giemsa (Fluka). Slides were washed in H_2_O and mounted with DPX solution.

### Granule preparation

Mast cell granules were isolated according to the method of Krüger *et al.* [26] with some modifications. The cells were resuspended in ice-cold balanced salt solution (BSS; 4 mM Na_2_HPO_4_, 2.7 mM KH_2_PO_4_, 150 mM NaCl, 2.7 mM KCl, 0.9 mM CaCl_2_, pH 7.2) supplemented with 0.175% bovine serum albumin (BSSA) at a cell density of 2–3×10^6^ cells/mL. Aliquots of 2.5 mL in 15×100 mm glass tubes were sonicated at 4°C in a bath sonicator (Selecta Ultrasons, JP Selecta, Spain) during 20 s. Sonicates were pooled and centrifuged at 200 g for 5 min at 4°C. The supernatant, containing the granules, was decanted and maintained at 4°C. The pellet, containing cellular debris and non-broken cells, was resuspended in BSSA, resonicated and centrifuged at 200 g for 5 min at 4°C. The resulting supernatant was combined with that obtained after the first round of sonication, and the complete suspension was then centrifuged at 2100 g for 20 min at 4°C. The resulting pellet, consisting of a crude granule fraction, was gently resuspended in 0.5 mL of ice-cold BSSA per 5–6×10^6^ original mast cells. The 0.5 mL was then layered on top of 2.5 mL of 90% Percoll solution, containing 150 mM NaCl, 2.7 mM KCl, 0,9 mM CaCl_2_ and 10 mM HEPES, and adjusted to a final pH of 7.2. Centrifugation was performed at 27,000 g for 20 min (Beckman Optima XL-90 ultracentrifuge, SW 40-Ti rotor; Beckman-Coulter, USA). After centrifugation, one single band containing the granules was obtained. This granule fraction was carefully collected and Percoll was removed by centrifugation at 100,000 g for 1 h at 4°C. Finally, the granules fraction was washed twice with ice-cold BSSA, immediately frozen with liquid nitrogen and stored at −80°C until analysis.

### Degranulation assay

Cells were stimulated to degranulate by two independent protocols, i.e. exposure to IgE/anti-IgE or to calcium ionophore A23187. For the IgE/anti-IgE protocol, cells (4×10^6^/mL) were preloaded for 6 h with anti-TNP mouse IgE (1 µg/mL) in medium. Next, sensitized cells were washed with phosphate buffered saline (PBS; 10 mM phosphate, 137 mM NaCl, 2.68 mM KCl (pH 7.4)), placed in Tyrode's buffer (10 mM HEPES, 130 mM NaCl, 5 mM KCl, 1.4 mM CaCl_2_, 1 mM MgCl_2_, 5.6 mM glucose, 0.1% (w/v) BSA, (pH 7.4)) at 4×10^6^/mL and stimulated with anti-mouse IgE (2 µg/mL) for 1 h. Stimulation with calcium ionophore A23187 was performed by treating the cells (4×10^6^/mL) with A23187 at a final concentration of 2 µM during 2 h in Tyrode's buffer. As a control, cells incubated during 2 h in Tyrode's buffer were used. To evaluate the extent of degranulation, the enzymatic activity of β-hexosaminidase in supernatants and cell pellets was measured as described in the next section.

### Hexosaminidase activity

β-hexosaminidase activity was assayed as an evaluation of the extent of degranulation following stimulation of cells under different treatments with IgE/anti-IgE or A23187, and also as an estimation of the intracellular and extracellular content of this enzyme in cell cultures under different treatments.

In the first case, after degranulation, the enzymatic activity of β-hexosaminidase in supernatants and cell pellets solubilized with 1% (v/v) Triton-X-100 in Tyrode's buffer was measured with *p*-nitrophenyl N-acetyl-β-D-glucosaminide in 0.05 M citric acid (pH 4.5) for 60 min at 37°C. The reaction was stopped by the addition of 0.05 M Na_2_CO_3_ (pH 10.7), and the release of the product *p*-nitrophenol was detected by absorbance at 405 nm. The extent of degranulation was estimated by calculating the percentage of *p*-nitrophenol absorbance in the supernatant *versus* total absorbance (supernatant + cell pellet solubilized in detergent).

To determine the intracellular and extracellular content of β-hexosaminidase in cell cultures (i.e. cells not stimulated for degranulation) we followed the assay described by Riederer *et al.* [27] with minor modifications. Briefly, 18-days old BMMCs were washed with PBS and resuspended in culture medium lacking phenol red. On day 21, secreted hexosaminidase activity was assayed in 0.5 mL media by addition of 125 µL 5 X hexosaminidase substrate buffer (0.5 M sodium acetate pH 4.4, 0.5% Triton-X-100, 5 mM *p*-nitrophenyl N-acetyl-β-D-glucosaminide). After 1 h at 37°C, reactions were stopped by addition of 0.6 mL of 0.5 M glycine, 0.5 M Na_2_CO_3_, pH 10. Release of *p*-nitrophenol was measured spectrophotometrically at 405 nm. One U of enzyme activity was defined as the amount of enzyme needed to hydrolyze 1 nmol per hour at 37°C. For intracellular hexosaminidase determination cells (3×10^6^) were lysed in 500 µL of 10 mM phosphate buffer pH 6, 150 mM NaCl, 0.5% Triton-X-100 and the lysate centrifuged at 100,000 *g* for 10 min at 4°C. Afterwards, the supernatant was recovered, further diluted (1/10) with the same buffer and the activity was assayed following the same procedure described above by mixing 50 µL with 575 µL of 1.1 X hexosaminidase substrate buffer.

### Amine determination

The intracellular content of putrescine, histamine, serotonin, spermidine and spermine was simultaneously determined by fluorimetry after separation of their dansyl derivatives by reversed-phase HPLC as previously described [28]. Amines in isolated mast cell granules were assayed by this method as well. In some experiments, extracellular contents of amines after cell degranulation (in Tyrode's buffer) were also determined by this HPLC method. Histamine and serotonin quantification in cell culture supernatants was performed by ELISA with the use of EIA-4616 (DRG, Germany) and ultrasensitive EIA BA10-5900 (Labor Diagnostika Nord, Nordhorn, Germany), respectively. Results were normalized to cellular numbers and are expressed as nmol of each amine/10^6^ cells.

### Transmission electron microscopy

Cells were fixed in 2% glutaraldehyde in 0.1 M cacodylate buffer, pH 7.2, supplemented with 0.1 M sucrose for 10 h. Cells were post-fixed in 1% osmium tetroxide in the same buffer for 90 min, dehydrated in graded series of ethanol and embedded with epoxy plastic Agar 100 (Agar Aids, Stansted, UK). Ultrathin sections were placed on Formvar-coated copper grids and constrated with 2% uranyl acetate and Reynolds Lead citrate. Analysis was performed in a Hitachi Electron Microscope at 75 kV. Ultrastructural features were observed in at least 200 cells.

### Western blot analysis

Protein expression levels for mMCP-6, mMC-CPA, acetyl-histone H3, COX-IV and M6PRBP1 were assayed by Western blot as described previously [28]. Primary antibodies were used at a dilution of 1∶2000 (anti-mMCP-6 and anti-mMC-CPA), 1∶1000 (anti-COX-IV and anti-M6PRBP1) and 1∶5000 (anti-acetyl-histone H3), and secundary antibody (HRP-conjugated anti-rabbit IgG; GE Healthcare) at 1∶5000. Normalization for sample loads was performed by re-probing membranes with the anti b-actin mouse monoclonal antibody described above.

### Histidine decarboxylase activity

Histidine decarboxylase (HDC) enzymatic activity was measured by following the release of ^14^CO_2_ from L-[^14^C]-labeled histidine as we have previously described [29]. Results are expressed as pmol of CO_2_ released/h/10^6^ cells.

### Total RNA isolation and quantitative PCR

Total RNA was isolated (4×10^6^ cells per extraction) following the protocol provided with the GenElute Mammalian Total RNA Miniprep Kit (Sigma-Aldrich) according to manufacturer instructions. RNA yield and purity were assessed spectrophotometrically at 260 and 280 nm, and only highly purified RNA (A^260^/A^280^ >1.95) was used. One microgram of RNA was treated with RNase free DNase (Promega, USA) and then reverse-transcribed using the iScript cDNA Synthesis Kit (Bio-Rad, USA) according to the recommendations of the manufacturers.

Quantitative PCR (qPCR) was performed in a final volumen of 25 µL, containing 1 µM of both primers, 1x SYBR Green supermix (Bio-Rad), and 1.5 µL of cDNA using Mx3000P machine (Stratagene, USA). The program profile used for all genes amplification was: 95°C for 10 minutes and 40 cycles of 20 seconds at 95°C, 15 seconds at 55°C and 30 seconds at 72°C. Plate reading was performed after each cycle. A melting curve was generated at the end of every run to ensure product uniformity. Hypoxanthine guanine phosphoribosyl transferase (HPRT) and beta-actin (ACTB) were used as housekeeping genes to compensate for differences in cDNA amount of M6PRBP1 between samples. The method used to calculate the relative amount of cDNA was described by Liu and Saint [30], and the relative efficiencies for individual amplification were calculated using the LinRegPCR program [31].

Primers sequences for M6PRBP1 and ACTB were obtained from the PrimerBank database [32] and were as follows: M6PRBP1 forward, 5′-ATGTCTAGCAATGGTACAGATGC-3′; M6PRBP1 reverse, 5′-CGTGGAACTGATAAGAGGCAGG-3′; ACTB forward, 5′-GGCTGTATTCCCCTCCATCG-3′; ACTB reverse, 5′-CCAGTTGGTAACAATGCCATGT-3′. Primers for HPRT were those used in a previous study [14] and were: HPRT forward, 5′-GATTAGCGATGATGAACCAGGTTA-3′; HPRT reverse, 5′-GACATCTCGAGCAAGTCTTTCAGTC-3′.

### Sample preparation for proteomic analysis, 2-D electrophoresis and protein identification

In brief (for complete description see Supplemental Data S1): for each sample, 20×10^6^ cells were lysed directly in 500 µL of 10% (w/v) TCA in acetone supplemented with 20 mM DTT, and proteins were allowed to precipitate for 1 h at −20°C. Next, samples were centrifuged for 10 min at 15,000 g at 4°C, and protein pellets were washed twice with acetone supplemented with 20 mM DTT and allowed to air dry. Subsequently, proteins were dissolved in 450 µL of rehydration solution at RT. Samples were clarified by centrifugation for 1 min at 15,000 g and were then applied to nonlinear pH 3–10 immobilized pH gradient strips. Next, isoelectric focusing was performed, and strips were then equilibrated for 15 min in SDS-equilibration buffer supplemented with 1% (w/v) DTT and for another 15 min with SDS-equilibration buffer supplemented with 2.5% (w/v) iodoacetamide. After equilibration, strips were applied to 10% SDS-PAGE gels. Electrophoresis was carried out at 2.5 W per gel during the first 30 min followed by 17 W per gel until complete. Proteins in the gels were fixed and stained using the silver staining procedure as described by Shevchenko *et al.* [33] and were stored at 4°C in 2% (v/v) acetic acid until mass spectrometry (MS) analysis. For gel-image analysis, gels were scanned at high resolution with a calibrated densitometer model GS-800 (Bio-Rad), and the PDQuest version 7.4 software (Bio-Rad) was used for detection of qualitative and quantitative alterations in protein spots. Spots of interest were excised from the gels, washed twice with water and were in-gel digested with porcine trypsin (Promega, Madison, WI) in essence as described by Shevchenko *et al.* [33]. The generated peptides were analyzed by MS using a 4700 MALDI-TOF/TOF mass spectrometer (Applied Biosystems, Foster City, CA). Protein identification was achieved by a combined strategy consisting of a peptide mass fingerprinting (PMF) search plus the MS/MS search of up to five peptide ions. Searches were performed using GPS Explorer™ software v 3.5 (Applied Biosystems) in non-redundant NCBI database of proteins using MASCOT searching engine (Matrix Science Ltd., London; http://www.matrixscience.com). Digestion of proteins in the spots, MS and PMF searches were performed either by the Proteomics Unit, SCAI, University of Córdoba, or by the Unidad de Proteómica, Edificio de Bioinnovación, University of Málaga.

### Statistical analysis

Statistical significance for amine determinations, HDC activity and β-hexosaminidase activity analysis was determined by the Student's paired samples *t*-test (two-tailed) using the GraphPad software. Values of *P<*0.05 were considered to be significant.

## Results

### Release of spermidine upon mast cell degranulation

**Figure 1:**
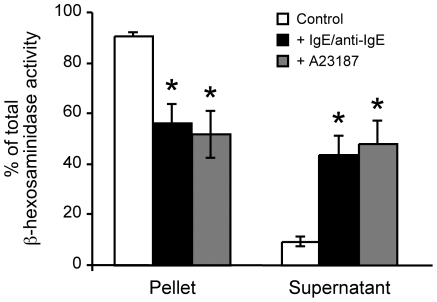
Activation and degranulation of BMMCs. Bone marrow precursor cells were cultured *in vitro* into BMMCs as described in Experimental Procedures. Cells (4×10^6^/mL) were stimulated to degranulate by exposure to IgE/anti-IgE or to calcium ionophore A23187 as described in Experimental Procedures. As a control, cells incubated during 2 h in Tyrode's buffer were used. Degranulation extent was monitored by determining the β-hexosaminidase activity in the cell pellets and supernatants, as described in Experimental Procedures. Results are expressed as percentages of total β-hexosaminidase activity and are means ± SEM of four independent experiments. *P<0.05 compared with control by Student's paired sample t-test (two-tailed).

**Figure 2:**
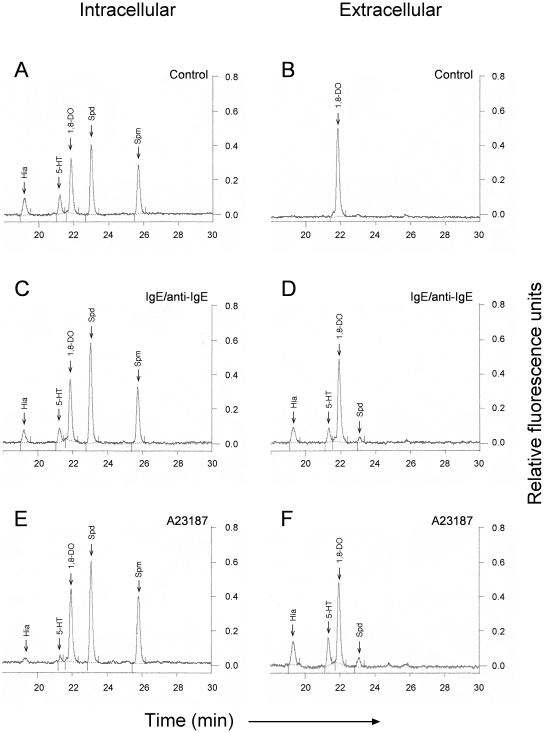
Intracellular and extracellular biogenic amines in BMMCs after degranulation. Bone marrow precursor cells were cultured *in vitro* into BMMCs as described in Experimental Procedures. Cells (4×10^6^/mL) were stimulated to degranulate by exposure to IgE/anti-IgE (C, D) or to calcium ionophore A23187 (E, F) as described in Experimental Procedures. As a control, cells incubated during 2 h in Tyrode's buffer were used (A, B). Amines were assayed in cell pellets (A, C, E) and supernatans (B, D, F) by HPLC as described in Experimental Procedures. Results shown are representative of three independent experiments. Put, putrescine; Hia, histamine; 5-HT, serotonin; 1,8-DO, 1,8-diaminooctane; Spd, spermidine; Spm, spermine.

To address the possibility that polyamines are associated with mast cell secretory granules, we first investigated if polyamines are released upon mast cell degranulation. Three weeks-old BMMCs were induced to degranulate by using two independent stimuli, treatment with IgE/anti-IgE or with calcium ionophore A23187. As shown in Fig. 1, both treatments induced the cells to degranulate, as judged by the reduced intracellular and increased extracellular β-hexosaminidase activity after addition of IgE/anti-IgE or A23187. The extent of degranulation calculated with respect to non-stimulated cells was about 30 and 40%, respectively, both values consistent with previous studies [13], [34]. Determination of biogenic amines both in the supernatants and cell pellets confirmed degranulation, as evidenced by the detection of histamine and serotonin in the supernatants (Fig. 2D and 2F) at the expense of a reduction of their corresponding intracellular levels (Fig. 2, compare 2C and 2E with 2A). In addition, a small but clearly distinguishable peak corresponding to spermidine was detected in the supernatants after both IgE/anti-IgE and calcium ionophore A23187 treatments (Fig. 2D and 2F), indicating release of this polyamine upon mast cell degranulation. Importantly, the magnitude of release of the different biogenic amines, including spermidine, was consistent with that of β-hexosaminidase (compare Fig. 2 with Fig. 1), with the stimulation by calcium ionophore A23187 being more effective than with IgE/anti-IgE.

### Detection of polyamines in isolated mast cell granules

**Figure 3:**
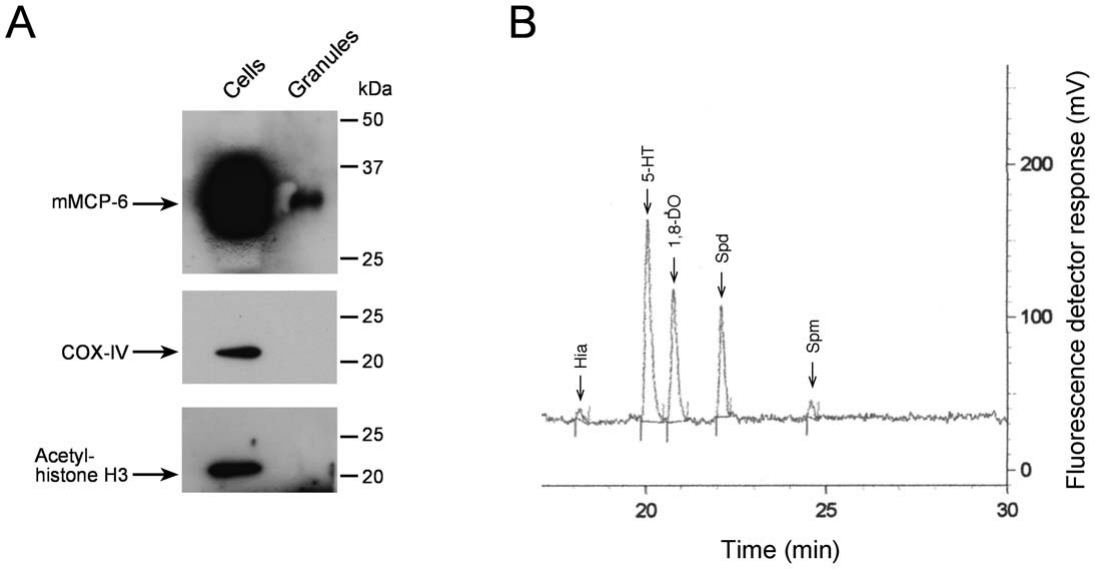
Qualitative detection of polyamines in isolated mast cell granules. Bone marrow precursor cells were cultured *in vitro* into BMMCs as described in Experimental Procedures and maintained until day 35. Granules were then isolated and detection of mMCP-6, COX-IV and acetyl-histone H3 was performed in total cell lysates and in the granule fraction (A) by Western blot. Amines were assayed in the granule fraction (B) by HPLC as described in Experimental Procedures. The chromatogram shown is representative of three independent experiments. Hia, histamine; 5-HT, serotonin; 1,8-DO, 1,8-diaminooctane; Spd, spermidine; Spm, spermine.

The release of spermidine upon mast cell degranulation supports the hypothesis that polyamines, at least spermidine, are associated with mast cell granules. To test this hypothesis further, we sought polyamines in isolated mast cell granules. Intact mast cell granules, i.e. membrane-bound secretory granules, have been isolated previously for different purposes from rat peritoneal mast cells [35]–[37] by using the method described by Krüger *et al.* [26]. Since, to the best of our knowledge, there is no specific procedure described for the isolation of intact granules from BMMCs, we used this method. Briefly, the method involves mild sonication of the cells followed by a low-speed centrifugation to remove cell debris, and a final purification of the granules using a Percoll density gradient. Typically, when rat peritoneal mast cells are used as starting material, two distinct bands are obtained after the Percoll gradient centrifugation: one upper band containing membrane-free granules and a lower band containing intact membrane-bound granules [26]. When applying this method to BMMCs, only a single band was obtained (not shown), most probably consisting of both intact and membrane-free granules. This fraction was carefully recovered and washed twice before further analysis, to eliminate any component not tightly bound. As shown in Fig. 3, the recovered fraction indeed contained mast cell secretory granules, as evidenced by the detection of tryptase mMCP-6, histamine and serotonin. Further, this fraction was free from major putative contaminations with other organelles, as we were unable to detect COX-IV or acetyl-histone H3, specific markers for mitochondrion and nucleus, respectively. Finally, and more striking, we detected a clear peak corresponding to spermidine and a weaker but discernible peak corresponding to spermine in the isolated granule fraction (Fig. 3B).

### Polyamine depletion leads to altered ultrastructure of mast cell granules

**Figure 4:**
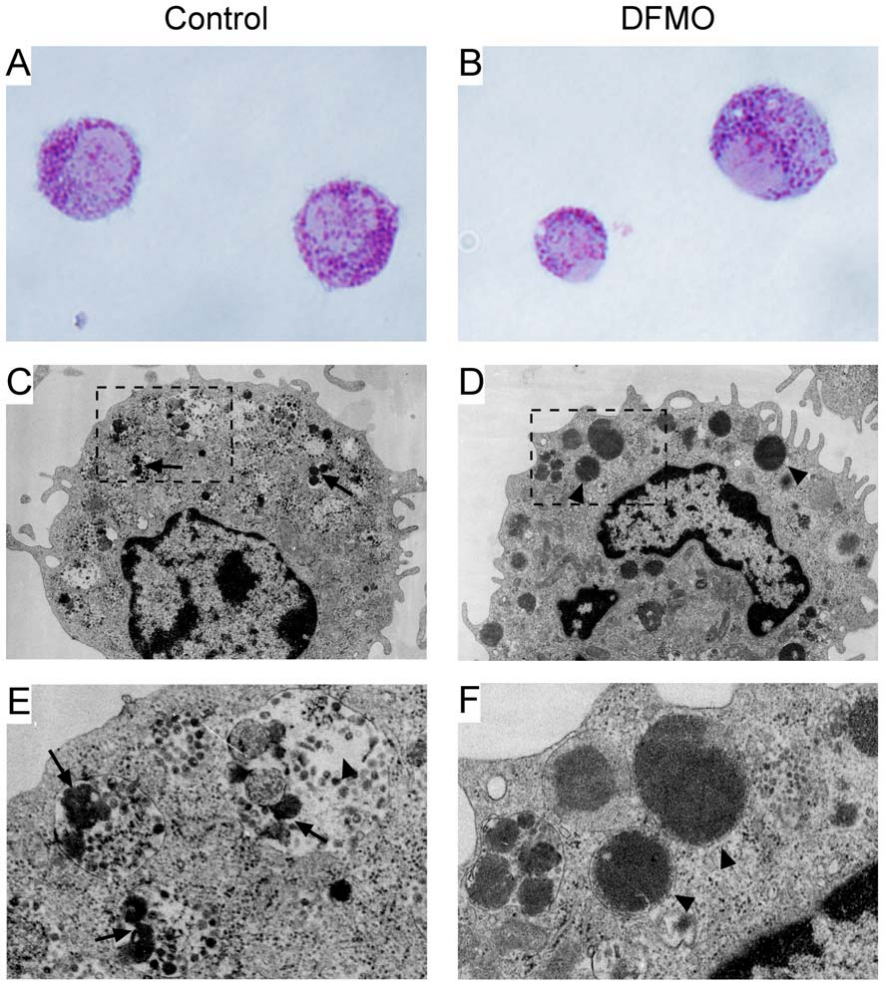
Secretory granule morphology of BMMCs generated in the absence or presence of DFMO. Bone marrow precursor cells were cultured *in vitro* into BMMCs as described in Experimental Procedures. On day 4, 5 mM DFMO was added to the culture medium and maintained for the rest of the culture time. After 3 weeks, untreated (A, C, E) or DFMO-treated (B, D, F) cells were examined by an optic microscope (May-Grünwald/Giemsa staining; A, B) or by transmission electron microscopy (C, D and in larger magnification E, F), as described in Experimental Procedures. Micrographs shown are representative from two independent experiments. Observe the abundance of densely stained secretory granules after May-Grünwald/Giemsa staining for both control (A) and DFMO-treated (B) cells. Note the presence of dense core formation (arrows in C and E) and electron translucent areas (arrowhead in E) in control cell granules. Note also that this subdivision is absent in granules of DFMO-treated cells, which instead contain evenly distributed, amorphous material throughout the entire granules, without dense core formation (arrowheads in D and F). Magnification: A, B, 950 x; C, D, 9,000 x; E, F, 27,900 x.

The previous results are compatible with the presence of polyamines in the mast cell granules, and we next investigated whether a depletion of polyamines may provoke any alteration in the morphology/appearance of mast cell granules. Polyamine depletion may be achieved by using DFMO, an irreversible inhibitor of ODC [38]. In a recent study we treated BMMCs with DFMO and obtained a large general reduction of both putrescine and spermidine [28], an effect in line with other works [39]. However, the possibility that polyamines are present in the mast cell secretory granules and the potential impact of polyamine presence on granule homeostasis has not been addressed previously. To specifically address these issues, BMMCs were generated in the presence of DFMO and cell morphology was analyzed by May-Grünwald/Giemsa staining and by transmission electron microscopy. Similarly to our previous study, an assessment of intracellular amine levels confirmed a large reduction of total polyamines after treatment with DFMO: putrescine was undetectable after the treatment and spermidine content was reduced by approximately 90% (Table 1). The reduction of total polyamines did not provoke a major effect on cell proliferation, a result not unexpected given the low proliferation rate of BMMCs (population doubling time of more than 4 days). Indeed, the cell numbers recorded prior to each medium change were only slightly reduced for the treated cells as compared with the untreated ones (not shown). As shown in Fig. 4A and 4B, the May-Grünwald/Giemsa staining did not reveal any major change in the staining properties of the granules after treatment of the cells with DFMO. Both control and DFMO-treated cells showed an abundance of densely stained secretory granules, suggesting a normal PG content. Transmission electron microscopy analysis, however, revealed striking differences in the ultrastructure of secretory granules. While non-treated control cells exhibited typical features of BMMC granules, i.e. a marked formation of dense core structures interspersed by electron translucent areas (Fig. 4C and in detail 4E), in DFMO-treated cells approximately 40% of the cells showed evenly distributed, amorphous material throughout the entire granules, without dense core formation (Fig. 4D and 4F). To rule out putative non-specific effects attributable to the inhibitor, a second control experiment was performed where cells were treated with DFMO in the presence of 100 µM putrescine, with the putrescine addition serving to reverse the effect of DFMO. Amine analysis in these cells demonstrated that, under these conditions, spermidine levels were restored to similar values as those of untreated control cells (Table 1). Moreover, an almost complete reversion of the effect observed in DFMO-treated cells was obtained, with approximately only a 7% of the cells showing the DFMO-induced phenotype (not shown). Taken together, these results suggest that spermidine is associated with mast cell granules, and indicate that polyamine depletion modifies secretory granule ultrastructure, the latter occurring without apparent alterations in the total PG content. Furthermore, the results support the hypothesis that polyamines are important for mast cell granule homeostasis.

**Table 1:** Polyamine levels in BMMCs generated in the absence or presence of DFMO or DFMO + putrescine.

Amine	Control	DFMO	DFMO + putrescine
Putrescine	0.2±0.05	ND	3.55±0.74*
Spermidine	0.37±0.04	0.03± 0.008**	0.44±0.01
Spermine	0.41±0.04	0.41±0.04	0.33±0.04

Bone marrow precursor cells were cultured *in vitro* into BMMCs as described in Experimental Procedures section. On day 4, 5 mM DFMO or 5 mM DFMO + 100 µM putrescine was incorporated or not (control) into the culture medium and maintained for the rest of the culture time. After 3 weeks, intracellular levels of polyamines were determined by HPLC as described in Experimental Procedures section. Results are expressed as nmol/10^6^ cells and are means ± SEM of at least three independent experiments.

*P<0.05;

**P<0.001 compared with untreated control cells by Student's paired sample t-test (two-tailed). ND: non detected.

### Effect of polyamine depletion on the storage of mast cell granule components

**Figure 5:**
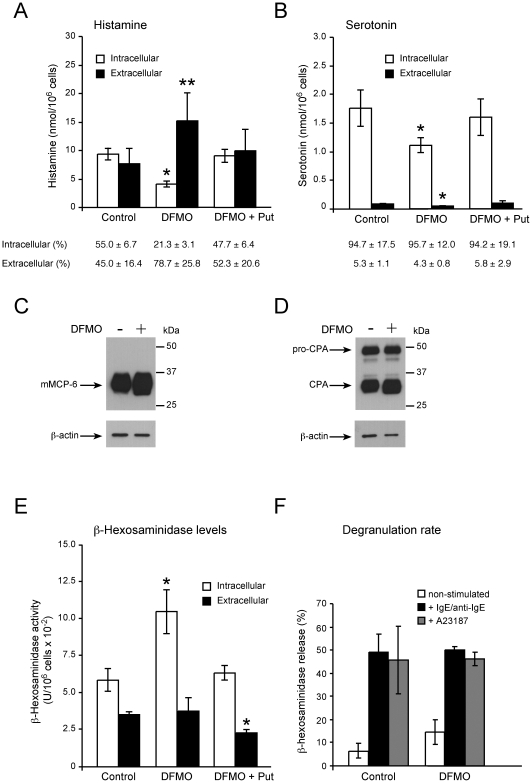
Effect of polyamine depletion on secretory granule component levels and degranulation ability in BMMCs. Bone marrow precursor cells were cultured in vitro into BMMCs as described in Experimental Procedures. On day 4, 5 mM DFMO or 5 mM DFMO + 100 µM putrescine was incorporated or not (control) into the culture medium and maintained for the rest of the culture time. After 3 weeks, intracellular levels of histamine (A) and serotonin (B) corresponding to 4×10^6^ cells were analyzed by HPLC. Extracellular levels of histamine (A) and serotonin (B) were assayed in cell culture media by ELISA. Protein levels for mMCP-6 (C) and mMC-CPA (D) were analyzed by Western blot (β-actin was used as a control of sample loads). Both intracellular and extracellular β-hexosaminidase content (E) and % release after the indicated conditions (F) were assayed as described in Experimental Procedures. The results displayed in A and B are means ± SEM of at least three independent experiments. The % of both intracellular and extracellular levels of each amine, calculated with respect to total values, are indicated beneath each corresponding plot in A and B. Western blots shown are from representative experiments. Results displayed in E and F are means ± SEM of three independent experiments. *P<0.03; **P = 0.06 compared with untreated control cells by Student's paired sample t-test (two-tailed).

Next, the effect of polyamine depletion on the storage of several mast cell secretory granule components, i.e. histamine, serotonin, mMCP-6, mMC-CPA and β-hexosaminidase was investigated. As shown in Fig. 5A and 5B, the intracellular levels of both histamine and serotonin were reduced by more than 40% after the DFMO treatment. However, while the DFMO treatment caused a substantial increase in extracellular histamine levels, resulting in an elevation of total histamine levels (Fig. 5A), extracellular serotonin levels were in contrast significantly decreased, leading to a reduced total amount of serotonin (Fig. 5B). Importantly, the observed effects were specifically attributable to the depletion of polyamines, since the addition of putrescine to the DFMO-treated cells reversed the effects (Fig. 5A and 5B). The reduced intracellular histamine content together with its elevated extracellular levels after the DFMO treatment, suggest an alteration of histamine storage as a consequence of polyamine depletion. In support of this hypothesis, histamine synthesis rate showed a slight trend to increase after the DFMO treatment, although not statistically significant (HDC enzymatic activity in pmol/h/10^6^ cells: control 8.31±1,76 *versus* DFMO 12.8±1.3; *P* = 0.24). On the contrary, the observed simultaneous reduction of both intracellular and extracellular levels of serotonin after the DFMO treatment, with an unchanged intracellular/extracellular ratio for the levels of this amine (see Fig. 5B), suggests that the DFMO treatment may lead to either a decreased synthesis or increased degradation of this amine, rather to an effect on the storage.

As shown in Fig. 5C and 5D, mMCP-6 and mMC-CPA protein levels showed only a mild trend to increase after the DFMO treatment. On the other hand, intracellular levels for the hydrolase β-hexosaminidase were substantially increased (Fig. 5E), an effect reversed with the addition of putrescine. Extracellular levels of this enzyme did not change after the DFMO treatment but, curiously, was significantly reduced upon addition of putrescine.

Finally, to investigate the possibility that polyamine depletion provokes any effect on the ability of the cells to degranulate, we analyzed the extent of degranulation after the DFMO treatment. However, the addition of DFMO did not affect the amount of β-hexosaminidase release in response to either IgE/anti-IgE or calcium ionophore A23187 (Fig. 5F), indicating that granule-contained polyamines do not influence the ability of BMMCs to undergo degranulation.

### Proteomic analysis

**Figure 6:**
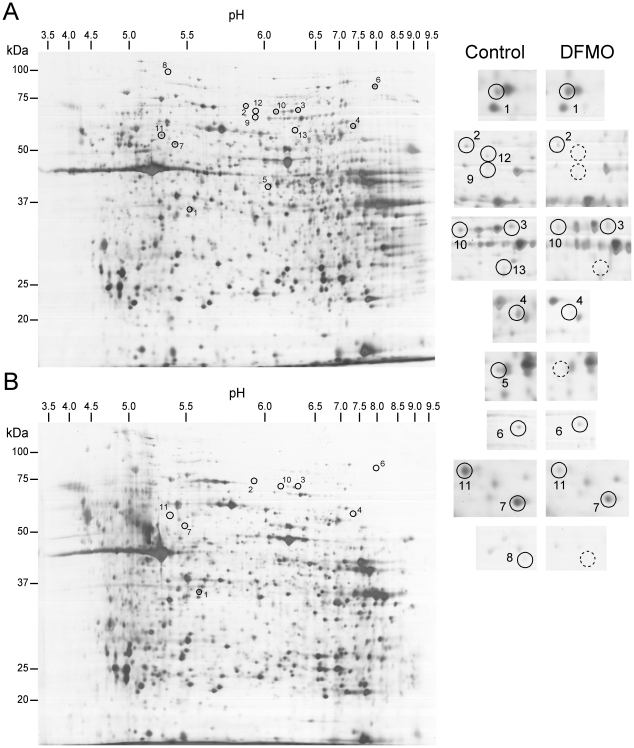
Representative 2D-gels for BMMCs generated in the absence or presence of DFMO. Bone marrow precursor cells were cultured *in vitro* into BMMCs as described in Experimental Procedures. On day 4, 5 mM DFMO was incorporated into the culture medium and maintained for the rest of the culture time. After 3 weeks, cells (20×10^6^) were taken and their proteins were extracted and separated by two-dimensional gel electrophoresis, as described in Experimental Procedures. Representative gels for untreated control BMMCs (A) and DFMO-treated BMMCs (B) are shown. Spots of interest are enclosed in circles and are numbered for their identification in Table 2. Magnification of the spots of interest are showed on the right. Discontinue-line circles indicate the putative position for spots whose detection was not achieved in gels corresponding to DFMO-treated cells. The position of the molecular weight standards is indicated at the left of each gel; the pH range is indicated at the top.

The results described above indicate that spermidine and spermine are associated with mast cell granules. Moreover, our data indicate that BMMCs with reduced total polyamine content exhibit aberrant secretory granules, accompanied by defective storage of histamine, decreased levels of serotonin and an increased content of β-hexosaminidase. To provide insight into the mechanisms underlying these observed effects, we explored putative proteins whose expression may be altered as a consequence of polyamine depletion. Proteins were separated by 2-D gel electrophoresis and the protein maps obtained were analyzed. Preliminary assays were conducted to determine the optimal experimental conditions for protein extraction from BMMCs and subsequent 2-D gel electrophoresis. Three different methods for protein extraction were tested: (i) use of the detergent CHAPS alone, (ii) CHAPS in combination with urea, and (iii) direct precipitation of proteins with TCA/acetone. The latter method proved to be the most adequate (see Supplemental Data S1, Fig. S1 and Table S1). Six samples corresponding to six independent BMMC cultures (3 controls and 3 DFMO-treated) were separated in 2-D gels (one sample per gel) and analyzed for protein spot differences. In general, protein patterns were very similar in all 6 gels obtained (not shown). However, a close image analysis revealed a number of protein spots whose intensities were remarkably changed after the DFMO treatment, and these were selected for protein identification by MS. Representative gels for both control and DFMO-treated cells are shown in Fig. 6, and proteins identified in the selected spots are listed in Table 2. In the majority of these cases, the DFMO treatment caused a markedly reduced intensity of the respective spot, with the exception of one spot, whose intensity was increased upon DFMO treatment.

**Table 2:** Identified proteins with altered expression levels in BMMCs generated in the presence of DFMO *versus* control BMMCs.

Spot No.^a^	Protein Name^b^	Relative expression^c^	UniProtKB acc. number	Comments (www.uniprot.org)
1	Pyruvate kinase M2 (PKM2; fragment)	↑ (3.8; *P*<0.01)	P52480	Glycolysis
2	Glycerol-3-phosphate dehydrogenase 2, mitochondrial (GPDH-M)	↓ (5.2; *P*<0.01)	A2AQR0	Gluconeogenesis
3	Phosphoenolpyruvate carboxykinase [GTP], mitochondrial (PEPCK-M)	↓ (7.1; *P*<0.02)	Q8BH04	Gluconeogenesis
4	Catalase	↓ (4.2; *P*<0.01)	P24270	Protection of cells from the toxic effects of hydrogen peroxide.
5	Heterogeneous nuclear ribonucleoprotein A/B (hnRNP A/B)	-	Q99020	Transcriptional repressor that binds to CArG box motifs, single-stranded and double-stranded DNA, and RNA
6	Splicing factor 1 (mZFM)	↓ (3.0; *P*<0,01)	Q64213	Necessary for the ATP-dependent first step of spliceosome assembly. May act as transcription repressor
7	Na(+)/H(+) exchange regulatory cofactor NHE-RF1 (NHERF-1 or EBP50)	↓ (3.6; *P*<0.01)	P70441	Scaffold protein that connects plasma membrane proteins with members of the ezrin-radixin-moesin family and thereby helps to link them to the actin cytoskeleton and to regulate their surface expression.
8	Major vault protein (MVP)	-	Q9EQK5	Required for normal vault structure. Vaults are multi-subunit structures that may act as scaffolds for proteins involved in signal transduction.
9	Dihydropyrimidinase-related protein 2 (DRP-2)	-	O08553	Necessary for signaling by class 3 semaphorins and subsequent remodeling of the cytoskeleton. Plays a role in axon guidance, neuronal growth cone collapse and cell migration
10	WD repeat-containing protein 1 (AIP-1)	↓ (5.2; *P*<0.02)	O88342	Induces disassembly of actin filaments in conjunction with ADF/cofilin family proteins
11	Mannose-6-phosphate receptor binding protein 1 (M6PRBP1 or TIP47)	↓ (4.0; *P*<0.01)	Q9DBG5	Transport of mannose 6-phosphate receptors (MPR) from endosomes to the trans-Golgi network

a) Spot number as indicated in Fig. 6.

b) Protein identification was accomplished by a combined PMF and MS/MS strategy, as described in Materials and Methods Section. Detailed information concerning searching results is provided in Table S2.

c) The minus sign (−) indicate absence of the corresponding protein spot in BMMCs generated in the presence of DFMO *versus* control BMMCs. Arrows (↑/↓) indicate statistically significant upregulation/downregulation of the corresponding protein spot in BMMCs generated in the presence of DFMO *versus* control BMMCs. Quantitative changes, either increases or decreases, are indicated in parenthesis as well as the level of significance according to a Student's t-test (fold; *P* value).

From Table 2 it is apparent that several of the proteins whose expression were altered upon polyamine depletion are involved in glucose metabolism: pyruvate kinase M2 (PKM2), glycerol phosphate dehydrogenase (GPDH-M) and phosphoenolpyruvate carboxykinase (PEPCK-M). We also detected a reduction in the levels of catalase, one of the major components that prevent the harmful effect of reactive oxygen species. There was also a downregulation of two proteins implicated as transcriptional repressors: heterogeneous nuclear ribonucleoprotein A/B (hnRNP A/B) and splicing factor 1 (mZFM). Two proteins with a structural or scaffold role that aid different functions including endocytic traffic and signal transduction, i.e. Na^+^/H^+^ exchange regulatory cofactor NHE-RF1 (NHERF-1) and major vault protein (MVP), were also decreased in samples corresponding to DFMO-treated cells. In addition, two proteins related to cell cytoskeleton remodeling, i.e. dihydropyrimidinase-related protein 2 (DRP-2) and WD repeat-containing protein 1 (AIP-1), were downregulated. Finally, and most striking, the DFMO treatment caused a downregulation of M6PRBP1, a protein clearly implicated in secretory granule formation. Proteins corresponding to spots no. 12 and 13, detected only in control cells, were not identified.

**Figure 7:**
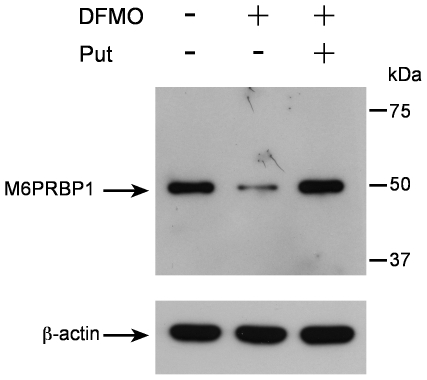
M6PRBP1 protein levels in BMMCs generated in the absence or presence of DFMO or DFMO + putrescine. Bone marrow precursor cells were cultured *in vitro* into BMMCs as described in Experimental Procedures. On day 4, 5 mM DFMO or 5 mM DFMO + 100 µM putrescine was incorporated or not (control) into the culture medium and maintained for the rest of the culture time. After 3 weeks, protein levels for M6PRBP1 were assayed in whole cell lysates by Western blot as described in Experimental Procedures. β-actin was used as a control of sample loads.

Based on the strong implication of M6PRBP1 in secretory granule formation, we validated its downregulation by an independent technique, i.e. by Western blot analysis. As shown in Fig. 7, protein levels for M6PRBP1 were drastically reduced in DFMO-treated cells, and this effect was polyamine-depletion specific as evidenced by the reversion of the DFMO effect caused by the addition of putrescine. In contrast, the levels of M6PRBP1-mRNA, as determined by quantitative RT-PCR, were not altered (not shown), indicating a post-transcriptional effect of DFMO-induced polyamine depletion on the expression of this protein.

## Discussion

Formation of mast cell secretory granules is a highly complex process absolutely essential for proper function of this cell type. Mast cell secretory granules contain, in addition to histamine/serotonin, cytokines, mast cell-specific proteases and serglycin PGs, also a large number of lysosomal enzymes. Therefore, these organelles have been termed “secretory lysosomes” [40]. The biogenesis of this subcellular compartment is dependent on the concerted contribution of both the exocytic and endocytic systems, with an array of signals that mediate the correct protein sorting to these organelles [41], many of which remain still unknown. In this process, serglycin PGs are of major importance, providing the skeleton necessary for the tight packaging of many compounds within the secretory granules [11], . Because polyamines have been described to aid the native conformation and packaging of other anionic biopolymers such as different RNAs and also DNA in the nucleus [17], and have been demonstrated to strongly interact with PGs [21], we undertook this study to address the possibility that polyamines are present in mast cell secretory granules, possibly interacting with granule PGs and thereby having a role in packaging the array of molecules that reside in these organelles.

The release of spermidine together with histamine and serotonin after triggering degranulation of BMMCs with either IgE/anti-IgE or A23187 calcium ionophore, provided the first evidence that polyamines, at least spermidine, are associated with the mast cell secretory granules. A second and more conclusive evidence was the detection of both spermidine and spermine in isolated mast cell granules. In addition, subsequent experiments showing profound alterations in the ultrastructure of BMMC granules as a consequence of polyamine depletion with DFMO, also support the presence of polyamines in the granules and suggest that these polycations are important for the correct biogenesis of these organelles. Secretory granules of BMMCs are typically organized into electron dense “core” regions interspersed by electron translucent areas and it was previously shown that the absence of serglycin PG led to impaired dense core formation, accompanied by a granule morphology with amorphous granule content [13]. Hence, serglycin holds a key role in the assembly of granule dense core material and this has proven vital for proper storage of a number of secretory granule compounds [11], [12], [14]. Interestingly, we here demonstrate that depletion of polyamines by treatment of BMMCs with DFMO causes a granule morphology that closely resembles that of serglycin-deficient granules [13], [43]. A likely explanation for this finding would be that DFMO treatment causes a reduction in the amount of serglycin PG present in the granules. However, staining of the BMMCs with May-Grünwald/Giemsa, a dye that preferentially stains for highly negatively charged PGs such as serglycin, did not reveal any reduction in staining properties following DFMO treatment, suggesting that DFMO does not act at the level of regulating serglycin storage. Further, DFMO treatment did not lead to a reduction in the storage of either MC-CPA or mMCP-6, granule compounds that were previously shown to be strongly dependent on serglycin for storage within the granules [11]. Thus, the most likely explanation for the alterations in granule morphology is that DFMO affects the process of dense core formation, independently of direct effects on serglycin. We may therefore hypothesize that optimal granule condensation in mast cells is a process that requires the concerted action of both serglycin and polyamines. It is important to note that the absence of serglycin does not seem to affect the level of intracellular polyamines in MCs [14]. Hence, although this study suggests that polyamines and serglycin both participate in maintaining granule homeostasis, polyamines and serglycin are not mutually dependent on each other for storage.

A striking finding was that DFMO treatment caused a marked reduction in the intracellular levels of histamine and serotonin. We showed in a previous study that both of these compounds are strongly dependent on serglycin for proper storage [14] and the present work thus demonstrates that both of these additionally require polyamines for maintenance of accurate intracellular levels. In the case of serotonin, we observed a substantial reduction of both the intracellular and extracellular levels, suggesting that the DFMO treatment might be modifying the rate of synthesis or degradation of this amine. By contrast, for histamine, the intracellular levels were reduced and the extracellular levels were increased, thereby indicating a diminished storage together with an augmented secretion of this amine due to the DFMO treatment. This conclusion is reinforced by the observed slight trend of increased HDC enzymatic activity, an effect in line with a previous recent study where we have described that the DFMO treatment leads to increased histamine synthesis in the heterogeneous cell population that make up the IL-3-driven bone marrow cell cultures after only 7 days [28].

Interestingly, DFMO treatment of the BMMCs also led to an increase in the intracellular content of β-hexosaminidase, an enzyme that was previously shown to be stored independently of serglycin [13]. At this stage we cannot explain the mechanisms behind this observation, although we may hypothesize that the reduction in other granule compounds may facilitate the accumulation of β-hexosaminidase. Alternatively, it cannot be excluded that polyamines exert direct effects on β-hexosaminidase, either by affecting the β-hexosaminidase protein content or by influencing its enzymatic activity.

Together, these results thus suggest that polyamines cause differential effects on individual mast cell granule components. Given the possibility that the population of mast cell secretory granules may be heterogeneous [41], , it is conceivable that polyamines cause distinctive effects on different subsets of granules. In line with this notion, a recent report has described that the antizyme inhibitor 2 (AZIN2; a protein that releases ODC from its inactive complex with antizyme) is expressed in mast cells, localizing in the serotonin-containing subset of granules (and not in tryptase-containing granules), where the authors propose that it could exert a role as a local regulator of polyamine biosynthesis, a process required for serotonin release upon IgE-mediated stimulation [45].

To gain insight into the mechanisms by which the depletion of polyamines with DFMO results in the observed BMMC phenotype, we conducted a proteomic analysis. We were able to detect several protein spots that were altered upon DFMO treatment. After MS identification, it is apparent that several of these do not exhibit an obvious connection with the machinery involved in the generation of mast cell secretory granules, e.g. GPDH-M, PEPCK-M, PKM2, all of these being involved in carbohydrate metabolism. Notably though, also previous work has shown that depletion of polyamines with DFMO affects the levels of enzymes involved in carbohydrate metabolism [46]. Also of note, limited evidence suggests a connection between glucose metabolism and the regulated exocytosis system: a reduced glycolysis rate occurring upon mast cell degranulation, an effect mediated by the ability of FcβRI to regulate PKM2 [47], and one report describing an association between the protein levels of PKM2 and annexin I and mast cell granule formation after treatment with nerve growth factor [48].

More importantly, we detected DFMO-induced alterations in a number of proteins that, because of their implication in either the endocytic or exocytic systems, could be contributing to the observed effects on granule storage/morphology. For example, we detected a DFMO-induced reduction of NHERF-1 and MVP, both proteins implicated in exo/endocytosis [49]–[54]. Also, we detected alterations in AIP-1 and DRP-2, both being involved in remodeling of the cytoskeleton. Indeed, the alteration of these proteins may contribute to the observed granule effects, considering the importance of cortical actin cytoskeleton dynamics for endocytic processes [55], [56]. Finally, we also detected a considerable downregulation in the levels of M6PRBP1. Since this protein plays a clear role in secretory granule formation, in particular as a component of the molecular machinery that sorts many granule compounds to the actual granules, its alteration could therefore provide the best direction, as compared to the other proteins detected, to further explore the mechanisms underlying the observed DFMO-induced effects on granule storage/morphology. Notably, several lysosomal hydrolases (including β-hexosaminidase) that are present both in conventional and secretory lysosomes, are modified during their biosynthesis by the addition of a M6P moiety. M6P is then recognized by M6PRs and these transmembrane receptors cycle between the trans-Golgi network (TGN) and late endosomes, targeting soluble proteins to conventional and secretory lysosomes [40]. M6PRBP1 (also known as TIP47) is one of the components responsible for the recycling of M6PRs back to the TGN [57], [58]. Conceivably, the observed reduction of M6PRBP1 upon DFMO treatment could thus impair M6PR-mediated targeting of granule compounds, providing a partial explanation for the effects seen on granule storage/morphology. Moreover, the observed alteration of β-hexosaminidase activity could be considered as a consequence of the impaired sorting of granule components. However, as mentioned previously, we cannot rule out that the DFMO-induced polyamine depletion alters either the β-hexosaminidase protein content or its enzymatic activity.

It is remarkable that almost 50% of the proteins that were reduced after DFMO treatment are implicated either in the endocytic or the exocytic system, clearly in line with a role of polyamines in regulating these processes. In support of this notion, previous studies have revealed polyamine depletion-induced effects related to these processes. For example, Hyvönen *et al.* [59], in a model of acute pancreatitis provoked by catabolism-induced polyamine depletion (i.e. by activation of polyamine catabolism in rats overexpressing spermidine/spermine-*N^1^*-acetyltransferase) described important changes in the ultrastructure of acinar cells, including the presence of early partially degranulated zymogen granules. Furthermore, Kanerva *et al.* [60] have recently described a critical role for AZIN2 in regulating the transport of secretory vesicles by locally activating ODC and polyamine biosynthesis. In this paper, the authors demonstrated selective fragmentation of the TGN and retarded exocytotic release of vesicular stomatitis virus glycoprotein upon RNAi-mediated knockdown of AZIN2 or DFMO-induced cellular polyamine depletion.

In summary, we here provide solid evidence that polyamines (i) are associated with mast cell secretory granules, (ii) are important for the correct maintenance of granule homeostasis (including appropriate storage of histamine) and our results (iii) suggest an important role for polyamines in regulating the complex machinery that leads to the formation of these organelles. Additionally, from a more general point of view, our findings together with those of other authors support the hypothesis of a critical function of polyamines in the processes of regulated exocytosis and endocytosis.

## Supporting Information

Figure S1 **Representative 2D-gels after BMMCs protein extraction using different methods.** BMMC proteins were extracted by the 3 procedures described in the Methods section, and were subjected to 2D-electrophoresis. For each procedure assayed, different amounts of proteins were tested (see Table S1). Gels shown correspond approximately to 20×10^6^ cells and were selected as being the best ones for each method as inferred in Table S1. A: lysis solution with CHAPS, 1000 µg of proteins. B: lysis solution with CHAPS + urea, 750 µg of proteins. C: proteins corresponding to 20×10^6^ cells precipitated directly with TCA/acetone. The position of the molecular weight standards is indicated at the left of each gel; the pH range is indicated at the top.
(TIF)

Table S1 Summary of the conditions used for the tested protein extraction methods and results obtained.
(DOC)

Table S2 Information regarding mass spectrometry identification of proteins in Table 2.
(DOC)

Data S1 Supplementary Material and Methods.
(DOC)
