# Supplementary figures and images for: Polyamines Are Present in Mast Cell Secretory Granules and Are Important for Granule Homeostasis
Source: PLoS One. 2010 Nov 30;5(11):e15071. doi: 10.1371/journal.pone.0015071 (PMC2994821; doi:10.1371/journal.pone.0015071)

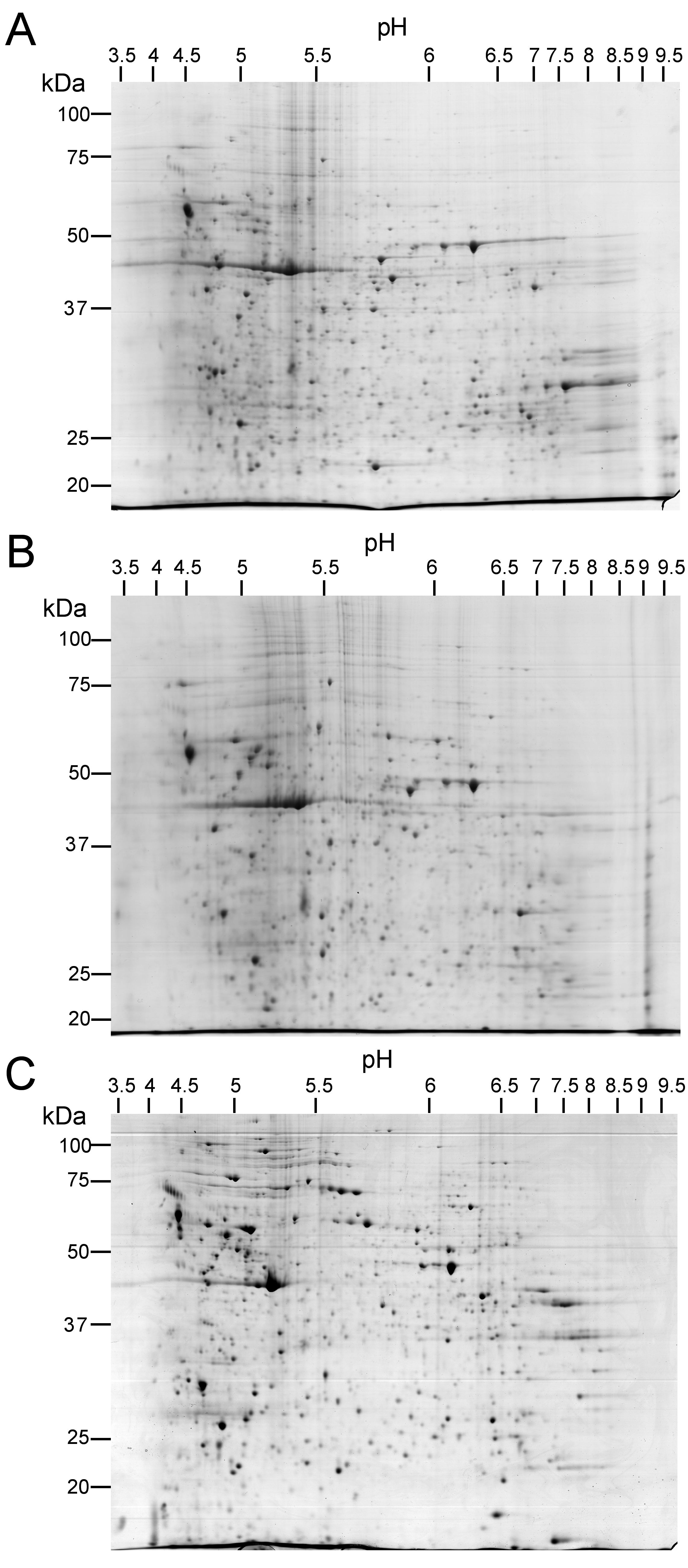

Supplement: Figure S1 — Representative 2D-gels after BMMCs protein extraction using different methods. BMMC proteins were extracted by the 3 procedures described in the Methods section, and were subjected to 2D-electrophoresis. For each procedure assayed, different amounts of proteins were tested (see Table S1). Gels shown correspond approximately to 20×106 cells and were selected as being the best ones for each method as inferred in Table S1. A: lysis solution with CHAPS, 1000 µg of proteins. B: lysis solution with CHAPS + urea, 750 µg of proteins. C: proteins corresponding to 20×106 cells precipitated directly with TCA/acetone. The position of the molecular weight standards is indicated at the left of each gel; the pH range is indicated at the top. (TIF) [file pone.0015071.s001.tif]
