# Supplementary material for: Polyamines Are Present in Mast Cell Secretory Granules and Are Important for Granule Homeostasis
Source: PLoS One. 2010 Nov 30;5(11):e15071. doi: 10.1371/journal.pone.0015071 (PMC2994821; doi:10.1371/journal.pone.0015071)
Supplement: Table S1 — Summary of the conditions used for the tested protein extraction methods and results obtained. (DOC) [file pone.0015071.s002.doc]

**García-Faroldi *et al.***

**Table S1**. Summary of the conditions used for the tested protein extraction methods and results obtained.

| **Extraction Method** | **Sample load^a^** | **No. spots^b^** | **Gel Quality^c^** |
| --- | --- | --- | --- |
| CHAPS | 750 μg prot. (14,5 x 10^6^ cells) | 1009 | ++ |
|  | 1000 μg prot. (19,5 x 10^6^ cells) | 1255 | ++ |
|  | 1335 μg prot. (26 x 10^6^ cells) | 919 | + |
| CHAPS + Urea | 750 μg prot. (21 x 10^6^ cells) | 911 | ++ |
|  | 1000 μg prot. (28 x 10^6^ cells) | 694 | + |
| TCA/acetone | 20 x 10^6^ cells | 1328 | +++ |

a) The equivalent numbers of cells for the CHAPS and CHAPS + urea methods are indicated in brackets

b) Number of detected spots according to PDQuest 7.4 software analysis.

c) Evaluated by the quality of spot focussing and contrast to the background (see Fig. S1).
