# Supplementary material for: Polyamines Are Present in Mast Cell Secretory Granules and Are Important for Granule Homeostasis
Source: PLoS One. 2010 Nov 30;5(11):e15071. doi: 10.1371/journal.pone.0015071 (PMC2994821; doi:10.1371/journal.pone.0015071)
Supplement: Table S2 — Information regarding mass spectrometry identification of proteins in Table 2. (DOC) [file pone.0015071.s003.doc]

**García-Faroldi *et al.***

**Table S2**. Information regarding mass spectrometry identification of proteins in Table 2

| **Spot No.^a^** | **Protein Name^b^** | **UniProtKB acc. number** | **Protein score/protein score CI %^c^** | **Matched peptides^d^** | **Sequence coverage (%)** | **No. peptides for MS/MS search^e^** | **Total ion score/total ion score CI %^f^** | **Theoretical  (kDa/pI)^g^** | **Observed  (kDa/pI)^h^** |
| --- | --- | --- | --- | --- | --- | --- | --- | --- | --- |
|  |  |  |  |  |  |  |  |  |  |
| 1 | Pyruvate kinase M2 (PKM2; fragment) | P52480 | 260/100 | 18 | 38 | 3 | 118/100 | 58.0/7.2 | 36.6/5.6 |
| 2 | Glycerol-3-phosphate dehydrogenase 2, mitochondrial (GPDH-M) | A2AQR0 | 435/100 | 28 | 35 | 5 | 202/100 | 83.0/6.0 | 74.0/5.8 |
| 3 | Phosphoenolpyruvate carboxykinase [GTP], mitochondrial (PEPCK-M) | Q8BH04 | 231/100 | 24 | 39 | 1 | 41/99.5 | 70.5/6,9 | 72.5/6.4 |
| 4 | Catalase | P24270 | 428/100 | 24 | 47 | 5 | 213/100 | 59.8/7.7 | 67.0/7.1 |
| 5 | Heterogeneous nuclear ribonucleoprotein A/B (hnRNP A/B) | Q99020 | 183/100 | 9 | 33 | 4 | 118/100 | 30.8/7.7 | 45.0/6.1 |
| 6 | Splicing factor 1 (mZFM) | Q64213 | 96/100 | 12 | 22 | 2 | 32/96.7 | 70.4/9.0 | 78.9/8.3 |
| 7 | Na(+)/H(+) exchange regulatory cofactor NHE-RF1 (NHERF-1 or EBP50) | P70441 | 391/100 | 24 | 68 | 4 | 134/100 | 38.5/5.6 | 60.5/5.5 |
| 8 | Major vault protein (MVP) | Q9EQK5 | 322/100 | 32 | 39 | 2 | 52/100 | 96.0/5.4 | 106.0/5.6 |
| 9 | Dihydropyrimidinase-related protein 2 (DRP-2) | O08553 | 204/100 | 19 | 40 | 4 | 59/100 | 62.5/5.9 | 72.6/5.9 |
| 10 | WD repeat-containing protein 1 (AIP-1) | O88342 | 140/100 | 16 | 26 | 2 | 40/99.5 | 73.0/8.7 | 72.6/6.1 |
| 11 | Mannose-6-phosphate receptor binding protein 1 (M6PRBP1 or TIP47) | Q9DBG5 | 122/100 | 11 | 38 | 3 | 49/99.9 | 47.3/5.4 | 62.0/5.4 |

a) Spot number as indicated in Fig. 6.

b) Protein identification was accomplished by a combined PMF and MS/MS strategy, as described in Methods Section.

c) Probability-based MOWSE score as obtained by PMF search achieved with MASCOT software. The confidence interval (CI) % for this search is also indicated (statistical significance CI % > 95).

d) Number of assigned peptides after PMF search.

e) Number of peptide ions fragmented and used for MS/MS search.

f) Total ion score is the sum of the individual ion scores obtained by the MS/MS searches of 1-5 peptide ions. CI % for this search is also indicated (statistical significance CI % > 95).

g) Calculated from the corresponding UniProtKB accession number with the complete (unprocessed) sequence.

h) Estimated from the position of the protein spot on the two-dimensional gel. *Mr* values were calculated according to the migration of *Mr* standards. *pI* values were determined by plotting each protein spot in the percentage of gel length *versus* pH graphs supplied by GE Healthcare Life Sciences.
