# Supplementary material for: Polyamines Are Present in Mast Cell Secretory Granules and Are Important for Granule Homeostasis
Source: PLoS One. 2010 Nov 30;5(11):e15071. doi: 10.1371/journal.pone.0015071 (PMC2994821; doi:10.1371/journal.pone.0015071)
Supplement: Data S1 — Supplementary Material and Methods. (DOC) [file pone.0015071.s004.doc]

**García-Faroldi *et al.***

**SUPPLEMENTAL DATA S1**

**SUPPLEMENTARY MATERIAL AND METHODS**

**Sample preparation for proteomic analysis**

Three different methods for protein extraction were tested including the use of the detergent CHAPS supplemented or not with urea, and the direct precipitation of proteins with TCA/acetone.

For the first method, 35 x 10^6^ cells were lysed in 100 μL of a lysis solution consisting of 50 mM tris-HCl pH 7.5 supplemented with 4% (w/v) CHAPS, 0.1 (w/v) SDS, 1 mM EDTA and a protease inhibitor cocktail (Sigma). Lysis was completed using a Branson 250 homogenizer (Branson Ultrasonics, USA). Then, samples were clarified by centrifugation for 10 min at 15,000 x *g* and proteins were quantified in supernatans with the Biorad Protein Assay kit (BioRad, Hercules, USA). Next, samples with different amounts of proteins (750, 1000 and 1335 μg) were brought to 75 μL with the same lysis solution, and were mixed with 375 µL of a 1.2 x rehydration solution consisting of 9.6 M Urea, 2.4 % (w/v) CHAPS, 0.336 % (w/v) DTT, 0.6 % (v/v) pH 3-10NL-IPG buffer (GE Healthcare Life Sciences) and 0.00288% bromophenol blue at RT. Subsequently, samples were subjected to 2-D electrophoresis (see below).

For protein extraction with a lysis solution containing CHAPS plus urea, 40 x 10^6^ cells were lysed with 100 μL of the same lysis solution described above, supplemented with 8 M Urea. Then, the same procedure described above was followed. Samples containing different amounts of proteins (750 and 1000 μg) were subjected to 2-D electrophoresis (see below).

In the case of protein extraction by precipitation with TCA/acetone, 20 x 10^6^ cells were lysed directly in 500 µL of 10 % (w/v) TCA in acetone supplemented with 20 mM DTT, and proteins were allowed to precipitate for 1 h at -20 ºC. Next, samples were centrifuged for 10 min at 15,000 x g at 4 ºC, and protein pellets were washed twice with acetone supplemented with 20 mM DTT and allowed to air dry. Subsequently, proteins were dissolved in 450 µL of 1 x rehydration solution (see above). Samples were clarified by centrifugation for 1 min at 15,000 x g and were then subjected to 2-D electrophoresis (see below).

**2-D Electrophoresis**

After protein extraction, all samples were applied to 24 cm nonlinear pH 3–10 immobilized pH gradient strips (Immobiline Drystrips pH 3-10NL; GE Healthcare Life Sciences), which were subsequently allowed to rehydrate for 12 h at 30 V (20 °C). Next, isoelectric focussing was performed at 20°C for 1h at 500 V followed by 1 h at 1000 V and 11 h at 8000 V, using a IPGPhor Isoelectric Focussing System (GE Healthcare Life Sciences). Strips were then equilibrated at room temperature for 15 min in SDS-equilibration buffer (50 mM Tris/HCl pH 8.8, 6 M Urea, 30% (v/v) glycerol, 2% (w/v) SDS and trace amount of Bromophenol blue) supplemented with 1% (w/v) DTT and for another 15 min with SDS-equilibration buffer supplemented with 2.5% (w/v) iodoacetamide. After equilibration, strips were applied to homogeneous 1 mm-thick 25.5 x 20 cm 10% SDS-PAGE gels prepared according to the Laemmli system (1). Molecular weight standards (BioRad) were applied to a well beside the strip. Electrophoresis was carried out at 2.5 W per gel during the first 30 min followed by 17 W per gel until complete. Proteins in the gels were fixed for 1 h in 10% (v/v) methanol 7% (v/v) acetic acid and stained with colloidal Coomassie for 24 h (2). When necessary, gels were destained with 50% (v/v) methanol 10% (v/v) acetic acid and silver-stained following the method of Shevchenko *et al.* (3), to achieve a higher sensitivity. Gels were stored at 4°C in 2 % (v/v) acetic acid until mass spectrometry analysis.

For gel-image analysis, gels were scanned at high resolution with a calibrated densitometer model GS-800 (BioRad), and the PDQuest version 7.4 software (Bio-Rad) was used for detection of qualitative and quantitative alterations in protein spots. Gel images were normalized to the total grey intensity. Significant quantitative changes in protein spots were determined according to a Student’s t-test.

Figure S1 shows the best quality gel obtained after testing each of the 3 protein extraction methods described above. Table S1 summarizes the conditions used for testing these methods and the results obtained. The method that provided the best gels, in terms of number of spots and image- and spot focussing quality, was that of the TCA/acetone direct protein precipitation.

**Mass spectrometry analysis and protein identification**

Mass spectrometry analysis and protein identification were carried out essentially as originally described by Shevchenko *et al.* (3) with the minor variations described by Maldonado *et al.* (4). Every spot of interest was manually excised with a blue tip and transferred to multiwell 96 plates. Next, spots were digested with modified porcine trypsin (sequencing grade; Promega) by using a ProGest (Genomics Solution) digestion station. Next, gel plugs were destained by incubation with 200 mM ammonium bicarbonate in 40% acetonitrile (ACN) at 37 °C (twice for 30 min), and then subjected to three consecutive dehydration/rehydration cycles with pure acetonitrile and 25 mM ammonium bicarbonate in 40% ACN, respectively. Finally, gel plugs were dried at room temperature for 10 min. Trypsin (20 μL), at a final concentration of 12.5 ng/μl in 25 mM ammonium bicarbonate was added to the dry gel pieces and the digestion proceeded at 37 °C for 12 h.

Peptides were extracted from gel plugs by 15 min incubation with 0.5% trifluoroacetic acid (TFA) 50% ACN, dried under vacuum and resuspended in 10 μL μl 0.1% TFA. One third of the sample was purified by ZipTip and deposited onto MALDI plate using the dry droplet method (ProMS, Genomic Solutions) using α-cyano hydroxycinnamic acid as matrix at a concentration of 5 mg ml^−1^ in 70% ACN/0.1% TFA. Mass spectrometry analysis was performed in a 4700 Proteomics Analyzer MALDI–TOF/TOF mass spectrometer (Applied Biosystems), in the m/z range 800 to 4000, with an accelerating voltage of 20 kV, in reflectron mode and with delayed extraction set to “on” and an elapsed time of 120 ns. Spectra were internally calibrated with peptides from trypsin autolysis (M+H+=842.509, M+H+= 2211.104). The 3 most abundant peptide ions were then subjected to MS/MS analysis, providing information that can be used to determine the peptide sequence.

Protein identification was achieved by a combined strategy consisting of a peptide mass fingerprinting (PMF) search plus the MS/MS search of up to five peptide ions. Searches (MS + MS/MS) were performed using GPS Explorer™ software v 3.5 (Applied Biosystems) in non-redundant NCBI database of proteins using MASCOT searching engine (Matrix Science Ltd., London; http://www.matrixscience.com) allowing the following parameters: taxonomy restrictions to Mammalia, one missed cleavage, 100 ppm mass tolerance in MS and 0.5 Da for collision-induced dissociation (CID) data, cysteine carbamidomethylation as a fixed modification and methionine oxidation as a variable modification. The confidence in the peptide mass fingerprinting matches (*P,* 0.05) was based on the MOWSE score (higher than 65) and confidence interval (CI) 99.8%, and confirmed by the accurate overlapping of the matched peptides with the major peaks of the mass spectrum. Only hits to *Mus musculus* were taken into consideration.

**References**

S1. Laemmli, U. K. (1970) *Nature* **227**, 680-685

S2. Neuhoff, V., Arold, N., Taube, D., and Ehrhardt, W. (1988) *Electrophoresis* **9**, 255-262

S3. Shevchenko, A., Wilm, M., Vorm, O., and Mann, M. (1996) *Anal Chem* **68**, 850-858

S4. Maldonado, A. M., Echevarria-Zomeno, S., Jean-Baptiste, S., Hernandez, M., and Jorrin-Novo, J. V. (2008) *J Proteomics* **71**, 461-472

**FIGURE S1 LEGEND**

Fig. S1. Representative 2D-gels after BMMCs protein extraction using different methods. BMMC proteins were extracted by the 3 procedures described in the Methods section, and were subjected to 2D-electrophoresis. For each procedure assayed, different amounts of proteins were tested (see Table S1). Gels shown correspond approximately to 20 x 10^6^ cells and were selected as being the best ones for each method as inferred in Table S1. A: lysis solution with CHAPS, 1000 μg of proteins. B: lysis solution with CHAPS + urea, 750 μg of proteins. C: proteins corresponding to 20 x 10^6^ cells precipitated directly with TCA/acetone. The position of the molecular weight standards is indicated at the left of each gel; the pH range is indicated at the top.
